# Early single-stage surgical revascularization of pulmonary artery in unilateral absence of a pulmonary artery

**DOI:** 10.1186/s13019-021-01481-3

**Published:** 2021-04-13

**Authors:** Wenlei Li, Li Ma, Shuliang Xia, Minghui Zou, Weidan Chen, Xinxin Chen

**Affiliations:** Heart Center, Guangzhou Women and Children’s Medical Center, Guangzhou Medical University, 9# Jinsui Road, Guangzhou, 510623 China

**Keywords:** Unilateral absence of a pulmonary artery, Revascularization, Surgery

## Abstract

**Background:**

This research aims to summarize the findings of the early single-stage revascularization of remnant pulmonary artery in unilateral absent intrapericardial pulmonary artery.

**Methods:**

We retrospectively analyzed the medical records of 10 patients with unilateral absent pulmonary artery, in which 7 were right and 3 were left, the median age and mean weight at surgery was 4 months and 5.6 kg, respectively. The patients received operation from January 2009 to June 2020.

**Results:**

Ten patients, 1 case associated with atrial septal defect, 2 cases with tetralogy of Fallot, and 1 case with aortopulmonary window. The mean diameter of the affected hilar pulmonary artery remnants was 3.14 ± 1.09 mm (1.6-5 mm), and the Z value was − 3.66 ± 1.86 (range, − 6.7 to − 1.75). All the patients received single-stage revascularization: tube graft interposition in 3 patients, autologous pericardial roll in 4, direct anastomosis in one, and main pulmonary artery flap angioplasty in the rest 3. No hospital deaths occurred. Mean follow-up in this cohort was 3.3 ± 1.9 years One case underwent percutaneous balloon dilatation due to new pulmonary artery stenosis. Nonetheless, the results were encouraging, symptoms have improved in all patients. The median Z value of the latest ipsilateral pulmonary artery diameter was − 1.88 (range, − 4.52 to − 1.35), a significantly improvement when compared to the preoperative value. The Z value of that in patients who using Gore-Tex tube increased relatively small.

**Conclusions:**

Single-stage pulmonary artery revascularization is effective at restoring normal antegrade flow to the affected lung, resulting in improved diameter of the PA, regression of pulmonary hypertension, and patient’s symptoms. Revascularization by using the autologous tissue or autologous pericardium may obtain a preferred result. The new pulmonary artery stenosis certainly will need to be addressed in the long-term follow-up.

## Background

Unilateral absence of a pulmonary artery (UAPA), also described as ductal origin of a pulmonary artery (DOPA), is a rare anomaly caused by the failure of the sixth aortic arch to connect with the pulmonary trunk during the embryologic development [1]. Most patients of UAPA often coexist with cardiovascular malformations such as tetralogy of Fallot (TOF), atrial septal defect, aortic arch constriction, subaortic stenosis and transposition of the great arteries, which could be diagnosed due to symptoms such as pulmonary hemorrhage, recurrent respiratory infection, pulmonary hypertension, and congestive heart failure. Conversely, approximately 30% of patients with UAPA has no associated cardiovascular anomalies, termed isolated UAPA, which are asymptomatic [2–5].

The absence of isolated pulmonary arteries was first described by Fraentzel in 1868 [6], followed by approximately 350 cases of UAPA reported in the world literature [7, 8], but there still lacks in clinical management for UAPA in infants and children.

Further, there are also limited literature on the treatment of revascularization of the affected lung. Currently, surgical repair involves connecting hilar pulmonary artery (PA) to the main pulmonary artery (MPA) by direct anastomosis or staged repair [9]. We summarized the experience of 10 cases of UAPA on the diagnosis and single-staged surgical treatment and the outcomes.

## Patients and methods

The study covered all UAPA patients who underwent one-stage surgical correction after diagnosis at Guangzhou women and children’s medical center from January 2009 to June 2020. The diagnosis of UAPA has been made according to preoperative examination, including chest X-ray, electrocardiogram, echocardiography, contrast-enhanced cardiac computed tomography (CT) and the intraoperative exploration. We excluded patients with these conditions: pulmonary atresia with major aorto-pulmonary collaterals and absence of intrapericardial pulmonary; agenesis of one lung; acquired pulmonary artery occlusion. By viewing medical records, echocardiograms and radiographic images and reports, the data are collected. The data available for research analysis includes age and symptoms at the time of presentation, diagnostic information, details of surgical procedures performed, complications, and outcomes (including growth and flow velocity of the affected PA). Mean follow-up in this cohort was 3.3 ± 1.9 years (range, 1 month to 5.6 years), Chest radiographs, echocardiograms, electrocardiograms were used in follow-up examinations. Cardiac computed tomography or catheterize angiography were performed if necessary. Current physical examination and symptoms information was obtained whenever possible. Data were analyzed using the Statistical Package for the Social Sciences (version.22.0; SPSS Inc) and presented as mean ± standard deviation and range, or median [Q1, Q3], as appropriate. Comparisons between pre-operative and post-operative data were made using the Wilcoxon signed-rank test. Ethical approval was waived by the institutional ethics committee of the Guangzhou women and children’s medical centerin view of the retrospective nature of the study and all the procedures being performed were part of the routine care.

## Results

### Patients characteristics

**Fig. 1:**
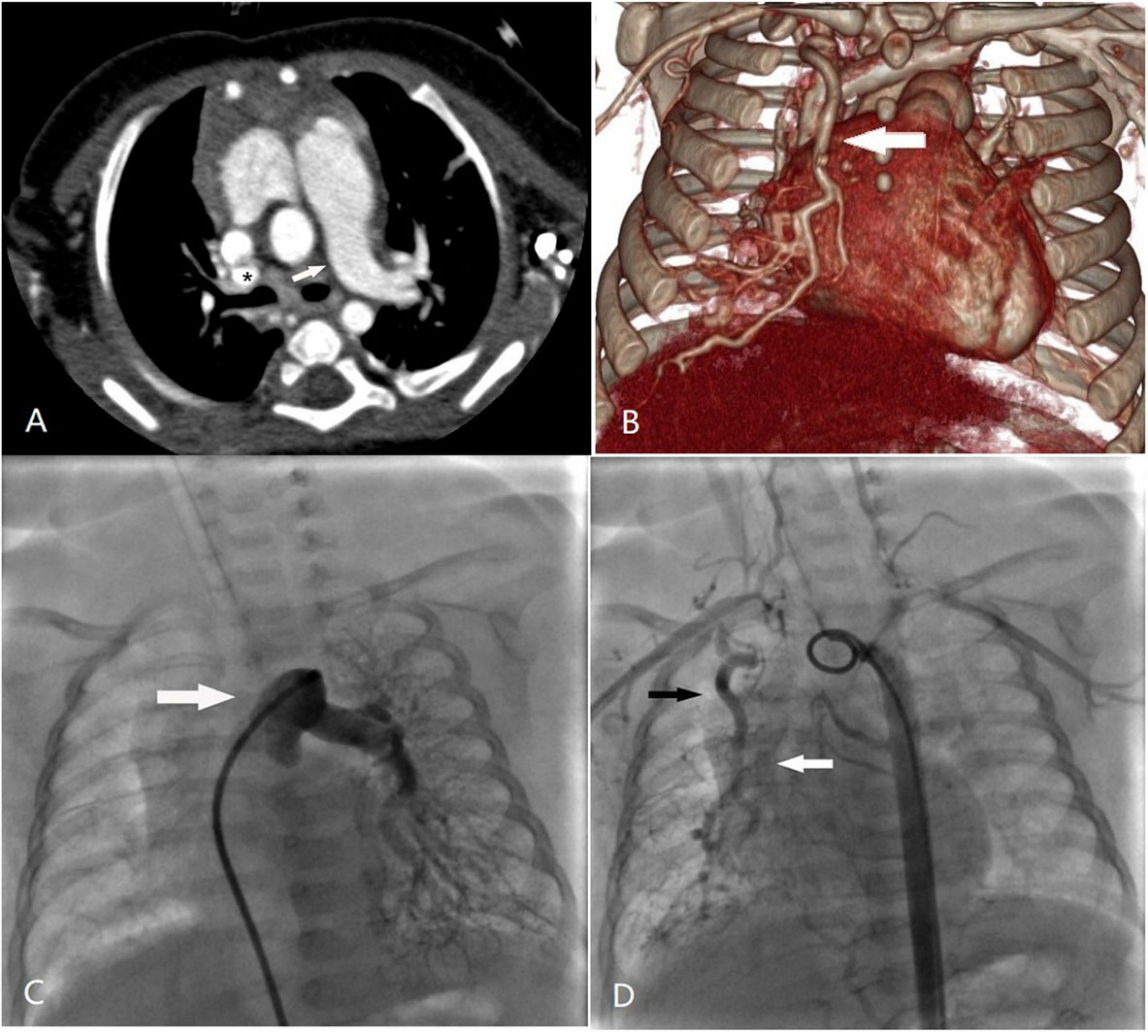
Images of a 2-month-old girl (patient No.5 in Table 1) who was diagnosed UAPA. **a** and **b** CT imaging; **c** and **d** cardiac catheterization. **a** Axis image on contrast-enhanced CT. The arrow shows the absence of the RPA. A small distal right PA in the hilum was marked by the asterisk. **b** Three-dimensional CT reconstruction. The arrow shows the abnormal thickened right internal thoracic artery. **c** Right PA was absence in the angiography of the pulmonary artery. **d** An abnormal vascular cluster from thickened right internal thoracic artery (black arrow) feeding into the distal right PA (white arrow). The aorto-pulmonary collateral arteries arose from descending aorta was thinly scattered

Surgical reconstruction of the PA in ten patients have been completed with 5 males and 5 females. The median age at the time of operation was 4 months (range, 16 days to 22 months). Mean body mass was 5.6 ± 1.8 kg. Detailed clinical characteristics were shown in Table 1. Two patients came with TOF had cyanosis, and one patient showed hemoptysis, while all the other children were found with the pre-cardiac murmur or respiratory distress. On chest radiography, it was found that the volume of the lungs on the ipsilateral side was reduced in 6 patients, the mediastinum shifted to the affected side, and the blood vessel markings were reduced. The echocardiogram indicated the main PA with a single branching PA. Eight patients without TOF showed a mean 4.4 ± 0.3 (range, 4.1–4.8 m/sec) of peak velocity of tricuspid regurgitation, which indicated the contralateral pulmonary arterial hypertension at the time of UAPA diagnosis. The CT showed absent of intrapericardial PA, the distal PA in the hilum and the blood supply of the affected lung. Cardiac catheterize angiography was performed on patient No,5, because the unsatisfactory anatomic delineation in CT images. Nine patients had UAPA affecting the lung contralateral to the aortic arch: right lung/left arch (*n* = 7), left lung/right arch (*n* = 2). Among the 8 patients, a patent ductus (*n* = 1) or a small stump (*n* = 7) was observed at the base of the brachiocephalic artery, which indicated the ductal origin of the abnormal PA. One had blood supply of affected lung from minor collaterals, and the other one’s right lung arterioles have connection with an abnormal vascular cluster from right internal thoracic artery, visualized by cardiac catheterize angiography (Patient No.5, Fig. 1). The isolated distal PA in the hilum of these patients was hypoplastic (3.14 ± 1.09 mm, range, 1.6-5 mm; Z value, − 3.66 ± 1.86; range − 6.7 to − 1.75). For the quantitative assessment of two TOF patients’ contralateral pulmonary artery, the Nakata index (NI) (mm^2^/m^2^), McGoon ratio were calculated according to previously published equations [10]. Values of NI (mm^2^/m^2^) and McGoon ratio in No.4 and No.10 patients were 205.9,0.62 and 397.7,1.69 respectively. Delay in diagnosis was infrequent.

**Table 1:** Clinical information for patients with unilateral absence of a pulmonary artery treated with revascularization of the pulmonary artery

No.	Sex	Age at operation	Diagnosis (arch sidedness)	Clinical presentation	Blood supply of affected lung	Diameter of remnant PA (mm) (Z value)	Intrapulmonary artery centralization techniques	F/U duration (y)	Latest diameter of affected PA (mm) (Z value)	Latest blood flow velocities of affected PA (m/s)
1	M	46 d	ARPA (left arch)	Murmur at birth	Brachiocephalic artery	3 (−3.01)	6 mm Gore-tex tube	5.6	5.7 (−2.99)	1.9
2	F	16 d	ARPA, ASD (left arch)	Respiratory distress at birth	Brachiocephalic artery	1.6 (−6.74)	5 mm Gore-tex tube	5.4	4.5 (−4.52)	1.6
3	F	6 mo	ARPA, PDA (left arch)	Murmur at birth	Minor aortopulmonary collaterals	2.2 (−5.44)	6 mm Gore-tex tube	5.2	5 (−4.27)	2.1
4	M	3 mo	ALPA, TOF (right arch)	Cyanosis	Brachiocephalic artery	1.9 (−5.97)	MPA flap angioplasty	4.1	6.8 (−1.4)	2.5
5	F	78 d	ARPA (left arch)	Respiratory distress at birth	Right internal thoracic artery	4 (−1.88)	7 mm autologous pericardial roll	4	4.5 (−1.43)	1.8
6	M	6 mo	ARPA, APW (left arch)	Respiratory distress at birth	Brachiocephalic artery	3.9 (−1.75)	10 mm autologous pericardial roll	3	6.8 (−1.59)	1.2
7	M	4 mo	ALPA, Obstructive emphysema of right lung (right arch)	Murmur at birth	Brachiocephalic artery	3.2 (−2.82)	Direct anastomosis	2.7	5.6 (−1.84)	2
8	F	4 mo	ARPA, AAOCA (left arch)	Respiratory distress at birth	Brachiocephalic artery	2.6 (−4.6)	9 mm autologous pericardial roll	1.3	5.4 (−2.12)	1
9	M	22mo	ARPA, PDA, BPD of right lung, ILD (left arch)	Hemoptysis	PDA	5 (−1.83)	10 mm autologous pericardial roll	1.2	6.9 (−1.35)	1.4
10	F	9 mo	ALPA, TOF (left arch)	Cyanosis	Brachiocephalic artery	4 (−2.59)	MPA flap angioplasty	0.1	4 (−1.91)	2.2

*AAOCA* Anomalous aortic origin of a coronary artery, *ALPA* absence of the left pulmonary artery, *APW* Aortopulmonary window, *ARPA* absence of the right pulmonary artery, *ASD* atrial septal defect, *BPD* bronchopulmonary dysplasia, *F* female, *ILD* Interstitial lung Disease, *M* male, *MPA* main pulmonary artery, *PA* pulmonary artery, *PDA* patent ductus arteriosus, *TOF* tetralogy of Fallot

### Surgical procedures

The operation was performed on the 10 patients immediately after their diagnosis had been made. Isolated UAPA was diagnosed in 6 patients. Coexisting cardiac malformations included atrial septal defect (1 patient), TOF (2 patients), aortopulmonary window (1 patient, type III of Mori [11]). Here we present the surgical procedures of reconstruction of the “neo” right pulmonary artery (RPA) for example, and same surgical techniques were also adopted in rebuilding the “neo” left pulmonary artery. Surgical exploration confirmed UAPA by a median sternotomy incision, and the main pulmonary artery and collateral vessel of the aorta, pulmonary artery and right pulmonary artery of affected lung were completely divided. After the collateral vessel was harvested from the aorta, cardiopulmonary bypass with moderate hypothermia (25–30 °C) was established by cannulating the ascending aorta, superior and inferior vena cava. For those patients who were associated with intracardiac malformation, the vena cava was snared, the ascending aorta was clamped, and cardiac arrest was obtained by infusion of antegrade 4 °C histidine-tryptophan-ketoglutarate cardioplegia solution through the aortic root before a complete repair was performed.

**Fig. 2:**
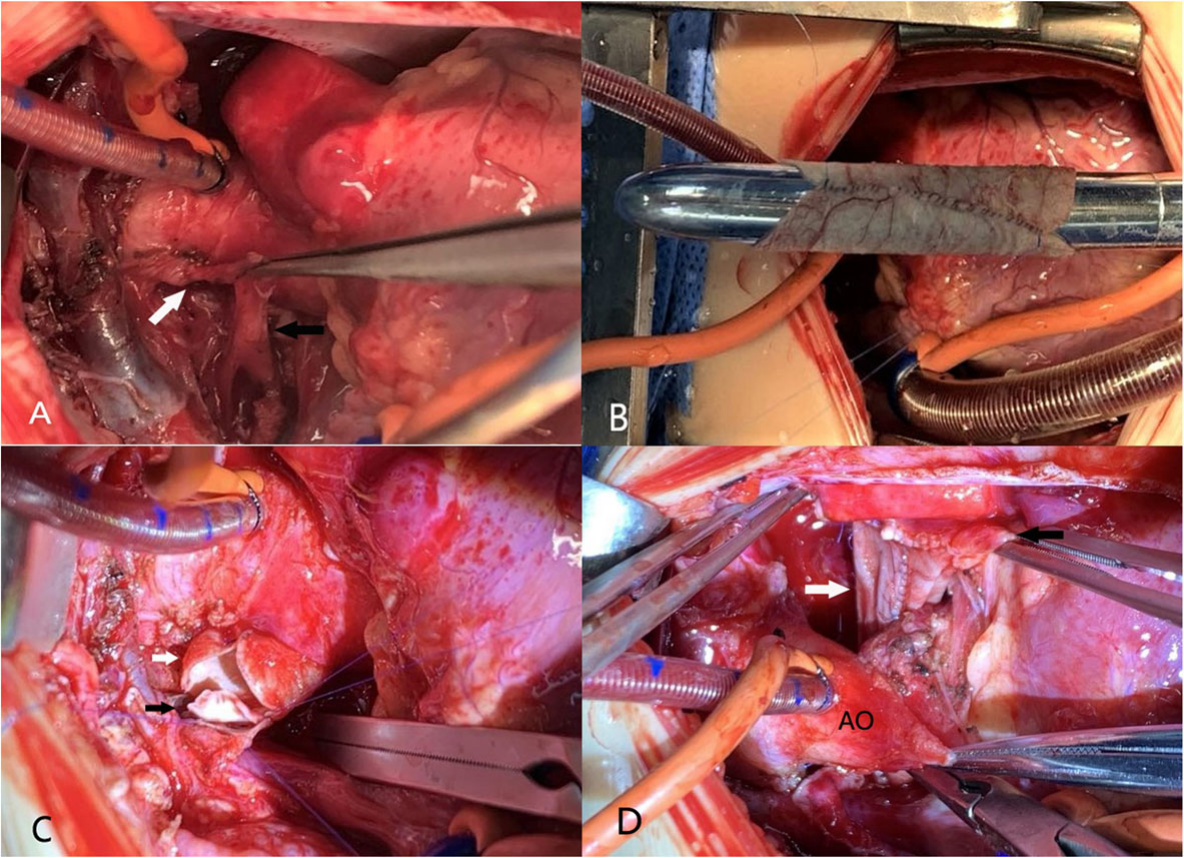
A 4-month-old girl (patient No. 8) with absence of the right PA. Intraoperative photo of a surgeon’s view. The patient’s head is to the left. **a** The distal right PA in the hilum (black arrow) and aortopulmonary collaterals (white arrow). **b** Hegar dilatator (9 mm)with autologous pericardial tube. **c**, **d** Anastomosis of the “tube” graft (white arrow) to the distal right PA (black arrow) and the main PA (black arrow)

A well-developed hilar PA could be connected to the posterolateral portion of the distal main PA trunk with a direct tissue-to-tissue anastomosis made with a running 7–0 prolene (Patient No.7). However, we could hardly perform this approach because the hypoplasia hilar PA. Two main approaches are used in intrapulmonary artery centralization. As for the first approach, if a wide distance between hilar RPA and MPA is observed, an autologous pericardial tube created with a running 7–0 prolene or a polytetrafluoroethylene (Gore-Tex, W. L. Gore&Associates, Inc) tube shall be taken to connect hilar RPA and MPA. In the cases herein, the authors directly reconnected the affected PA with a polytetrafluoroethylene tube graft in 3 patients (5 mm in 1 patient, 6 mm in 2 patients), whereas an autologous pericardial roll was applied in 4 patients (7 mm, 9 mm in 2 patients, separately, 10 mm in 2 patients) (Fig. 2). As for the second approach, if the “neo” PA posterior wall can be constructed by directly anastomosis the main PA flap and the hilum vessel, an autologous pericardium patch was used to augment the anterior surface of the “neo” PA from the hilum all the way back to the main PA. (Patient No.4, No.10) During these two approaches, the ascending aorta was not transected. The neo-PA was then placed under the ascending aorta. The mean cardiopulmonary bypass time was 69.7 ± 34.4 min (31–143 min).

### Acute outcomes and complications

There were no hospital deaths in those 10 cases. One patient with tetralogy of Fallot and absence of the left pulmonary artery occurred pulmonary hemorrhage after surgery (Patient No.4). The high-frequency oscillatory ventilation was applied for 5 days. A sternal debridement was performed 22 days after the first operation due to the infected median sternotomy wound (Patient No.6). One patient was treated with inhaled iloprost (Ventavis) 3 days for the control of acute pulmonary hypertension (Patient No.2), which might occur secondary to protamine administration when reversing heparin at the end of surgery. For the entire cohort, the median length of intensive care unit stay was 2.5 [2, 4] days (range, 1 to 14 days) and the mean duration of hospitalization was 18.2 ± 8.4 days (range, 5 to 34 days). To avoid thrombosis inside the “neo” branch PA, daily antithrombotic treatment with aspirin was prescribed in 6 months after the operation.

### Follow-up outcomes

No patient died during follow-up session. All patients were asymptomatic and demonstrated class I in the New York Heart Association (NYHA) classification.

**Fig. 3:**
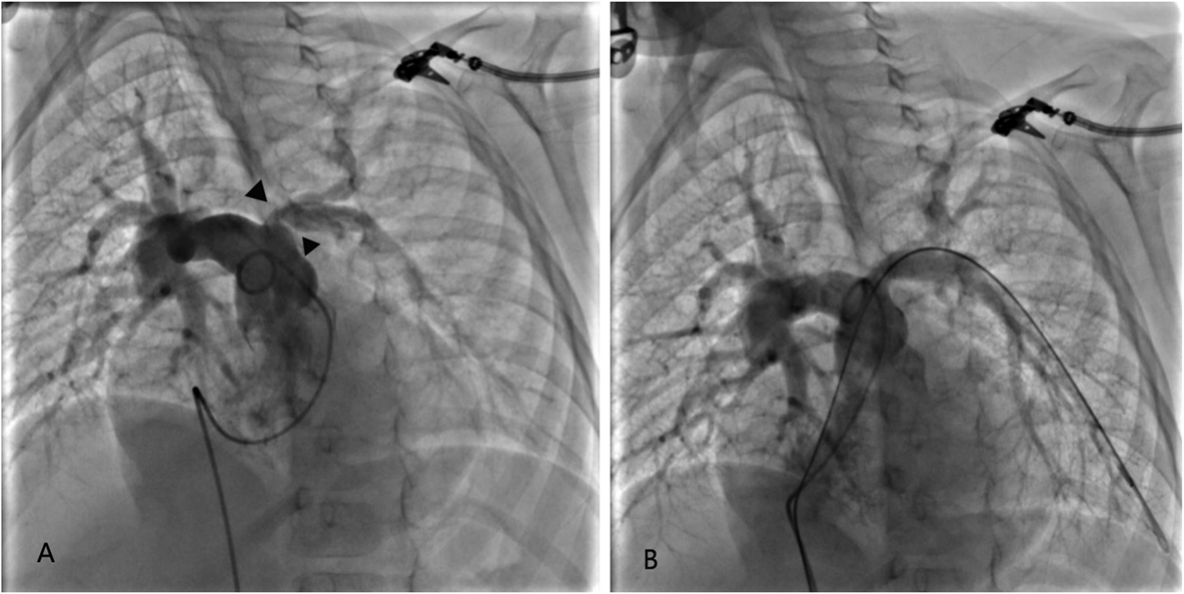
Anteroposterior pulmonary arteriogram in patient No.4 demonstrates left (black triangle) pulmonary opening stenosis (**a**) and enlargement after balloon dilation (**b**). The left pulmonary artery perfusion had improved

The left PA with stenosis at the anastomosis was found in patient No. 4 during serial follow-ups. Nonetheless, recurrent respiratory infections or exercise intolerance were not found in him. Yet, N-terminal pro-brain natriuretic peptide concentration > 500 pg/mL and high voltage of the right ventricle on echocardiography were found. We performed transcatheter balloon angioplasty at 41 months after surgery. The diameter of the focal stenosis increased from 1.8 mm to 6.8 mm, and the mean blood flow velocity decreased to 2.5 m/s. The main pulmonary arterial systolic pressure significantly decreased from 53 mmHg before transcatheter balloon angioplasty to 35 mmHg immediately after the intervention (Fig. 3).

**Fig. 4:**
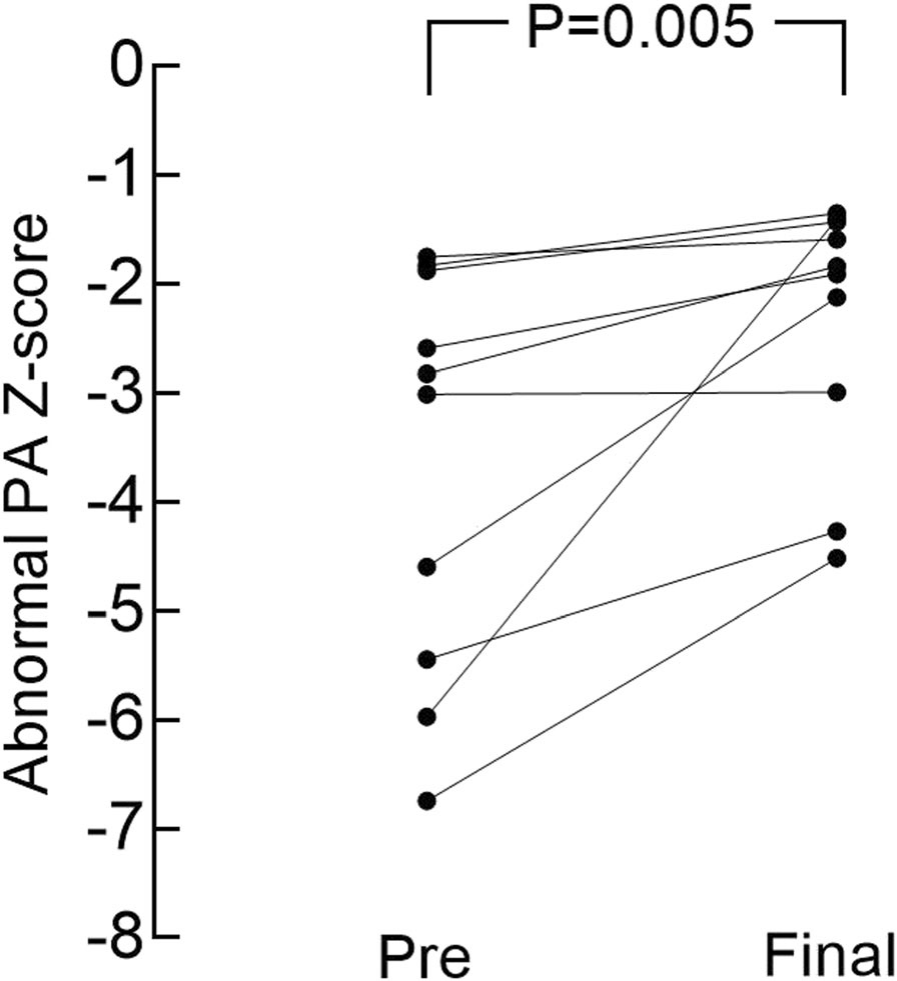
The effect of revascularization for the abnormal pulmonary artery. The Z-value of the affected PA significantly increased from − 3.66 ± 1.86 before surgery to − 2.34 ± 1.18 at latest follow-up

Serial echocardiographic findings showed no increase in flow velocity of the patient’s “neo” pulmonary artery. The blood flow velocities at the “neo” pulmonary artery were 1–2.5 m/s (Table. 1). At the latest follow-up, the mean size of the affected PA was 5.81 ± 1.07 mm (range, 4 to 7.4 mm), the median z-score of that was − 1.88[− 2.77,-1.47], range from − 4.52 to − 1.35), significantly improved when compared to the preoperative value. (Fig. 4). The Z value of the latest ipsilateral pulmonary artery in NO.1,2,3 patient who received operation of neo-pulmonary artery reconstruction at an early age by using Gore-Tex tube increased relatively small. (Table 1) There was mild or no tricuspid valve regurgitation with no evidence of pulmonary hypertension (defined as peak tricuspid regurgitation velocity < 2.8 m/sec). None of these patients had pulmonary hypertension preoperative that required long-term therapy.

## Discussion

UAPA is a rare congenital anomaly an incidence of approximately 1 in 200,000 first described by Fraentzel [6, 12]. Embryologically speaking, the proximal pulmonary artery branches develop from the proximal sixth aortic arch. UAPA occurs when the failure involution of the ipsilateral proximal sixth aortic arch with absence of the native PA and the ductus arteriosus to the hilar PA [1]. This lesion shall be isolated or associated with other congenital heart malformations [13].

UAPA indicates a wide spectrum of presenting symptoms, and reports in the literature show that about 12.9–30% of patients could be asymptomatic [7, 14]. The most common clinical symptoms include recurrent respiratory infection, and exertional dyspnea, while UAPA may present hemoptysis in 18 to 20% of patients with advancing age [7, 12]. The reason of hemoptysis is generally perceived to be caused by excessive collateral circulation with age [15]. In this study, patients were mostly diagnosed in young ages, and found with pre-cardiac murmur or respiratory distress. One patient presenting hemoptysis, who aged nearly 2 years old.

The diagnosis of UAPA may be missed in early aged patients. The chest X-ray shows the mediastinal displacement and decreased vascular markings on one side. An experienced sonographer could be able to find the diminished branch PA, calculate pulmonary artery pressure by measuring tricuspid regurgitation velocity and the abnormal lung blood supply. The images of CT could accurately show the intra-pulmonary artery distribution and the blood supply of the affected lung. The blood supply of the affected pulmonary artery includes bronchial, intercostal, subclavian, or internal thoracic arteries [16]. Eight of 10 patients had evidence of ductal arterial supply from brachiocephalic artery to the disconnected pulmonary artery. There is a relationship between the sidedness of the aortic arch and the absent of intrapericardial PA, which supports the findings of Pfefferkorn [1] and Mery’s cohort [9]. Cardio-angiography and magnetic resonance imaging also could provide hemodynamic data and accurate anatomic delineation to confirm the diagnosis and show the distal PA in the hilum [16].

Several literature recommended an early diagnosis and surgical intervention for UAPA. In this cohort, all the patients without TOF showed contralateral pulmonary hypertension at the time of diagnosis. Pulmonary hypertension may result from blood flow directed away from the absent pulmonary artery to the contralateral pulmonary artery. Increased blood flow leads to shear stress within the endothelium, with subsequent release of vasoconstrictive compounds. Early re-establishment of pulmonary blood flow may allow the affected lung to develop more normally and improve pulmonary hypertension [17]. At present, there is no consensus on the optimal age for surgical correction and the surgical approach. The surgical approaches vary from connection of the affected branch PA to the main PA to temporary palliation with ductal stent or creation of an aortopulmonary shunt with definitive correction later [18]. Murphy et al. [19] had performed staged surgical reconstruction of systemic shunt to the affected pulmonary artery on 7 patients at 3 months of age or younger, and concluded that early staged reconstruction could ensure subsequent growth of the pulmonary artery. Welch et al. [20] reported a direct anastomosis of right pulmonary artery and main pulmonary artery in a neonate and a 3-month-old infant with UAPA. By performing main pulmonary artery flap angioplasty or tube graft interposition to construct neo-PA in a series of 15 patients aged 1 day to 38 months, Kim et al. [21] concluded early one-stage surgical reconstruction and following transcatheter intervention on 11 patients improved pulmonary artery growth and lung perfusion. Mery et al. [9] published a case series with ten patients (median age: 2 years, range: 3 days to 9 years) who underwent one- and two-staged surgical repair of UAPA. Following PA centralization with interposition grafting using pericardial roll and anterior pericardial augmentation, four out of five patients required interventional procedures with balloon angioplasty and/or stent implantation during an average follow-up of 2 years. At present, there is still a controversy about the staging of surgery. The implementation of ductal stent or an aortopulmonary shunt may not only increase the preload of the left ventricular system, but also cause bilateral pulmonary hypertension. Heart failure treatment and pulmonary hypertension therapy are required [22].

Our series includes patients aged from 16 days to 22 months old at presentation, and these patients were in early ages when accepted the operation. We have performed single-stage collateral artery revascularized in 10 patients, who have shown accessible remnant PA at the hilum, found by CT, and the operations created superior outcomes. In this study, only one case achieved direct anastomosis, and the remaining 9 cases were indirectly matched due to the large distance and high tension. Direct anastomosis may achieve satisfactory results, and it could be done in newborns and small babies. Nevertheless, direct anastomosis cannot be applied in most cases, the duct-related tissue of the proximal end of affected branch pulmonary artery must be adequately resected to avoid stenosis or aneurysm formation. The cohort used two main surgical techniques for neo-PA reconstruction. Among them, 3 patients used Gore-Tex tube, and the remaining patients used the autologous tissue or autologous pericardium. As the child grows, the size mismatch between the graft and the native pulmonary artery shall develop, and follow-up surgery should be performed in future. More satisfactory results are received in the rest 7 patients. It is more ideal to use the autologous tissue or autologous pericardium in revascularization than any other prosthetic tubes. According to reports, the use of autologous tissue or autologous pericardium in revascularization is resistant to infection and calcification, and may have the potential for extensibility and growth [23]. The main problem with this technique comes with the part of the tissue sutured restenosis, which is caused by shrinkage of the scar tissue. The balloon angioplasty was performed on No.4 patient during the follow-up, whose reconstruction technology of neo pulmonary artery was MPA flap angioplasty.

Meanwhile previous report recommended transection of the aorta for better visualization when creating an anastomosis between the right and the main pulmonary arteries augmented anteriorly by a pericardial patch [24]. Moreover Moreno-Cabral et al. [25] reported one case that an autologous pericardial roll was placed in front of the superior vena cava and ascending aorta. With 4 years of follow-up, it was found that the anastomotic site stenosis occurred, which may be caused by compression of the ascending aorta. In our patients, the aorta and aortic arch were separated completely and retracted, a good visualization could also be obtained, and the tube was placed behind the superior vena cava and the ascending aorta to avoid the excessive tension and distortion of the neo-PA.

In the last review, there was no significant localized pulmonary artery stenosis found in these patients. All these patients showed significant growth of the affected PA. The Z value of the last ipsilateral pulmonary artery diameter significantly improved when compared to the preoperative value. In addition, no reoperation has been performed owing to the short follow-up periods.

UAPA combined with TOF is more complicated. The first successful complete repair of this defect was published by Sherrick [26] and the operative mortality in the past was 44 to 48% [27]. Numerous studies have reported the repair of this defect without revascularization of the affected lung. Bockeria, L. A. et al. [28] concluded a result of hospital mortality of 5% in 37 patients, they suggested the NI and NI Z-score should be greater than 200 mm^2^/m^2^ and − 4 for a successful complete repair, or palliative intervention was required. Yang, T. et al. [29] reported a good follow-up results with no deaths in 17 patients. They also suggested using the NI and NI Z-score as a criterion to evaluate the existing pulmonary artery. Meanwhile, few studies strongly suggested simultaneous or staged revascularization of the affected lung. There were three patients with TOF underwent ipsilateral Blalock–Taussig shunt as the initial surgery followed by complete repair and one patient accepted single-stage correction in Kim’s cohort [21]. There were 2 UAPA patients associated with TOF in this cohort. The NI and McGoon ratio meet the criterion which the previous study prescribed [28]. The primary correction was performed with the revascularization of the affected PA. Only one patient with a lower NI and McGoon ratio occurred pulmonary hemorrhage after surgery (Patient No.4).

There are several limitations to this study. First, for it is a retrospective design of a single center, the long-term results are unclear. The anomaly is rare, so the sample size is small. It may reduce the reliability of statistical differences between certain parameters. Due to the lack of chest radiography and pulmonary vein wedge angiography data and lung perfusion scan image, the author cannot ideally distinguish the prognosis of the case. Finally, the surgery in this study was performed by three surgeons, and differences in surgical techniques may affect the results.

## Conclusion

In conclusion, UAPA is a rare anomaly that could be often be misdiagnosed. An early single-staged surgical rehabilitation is recommended to ensure subsequent growth of the pulmonary artery if the distal PA in the hilum can be found and prevent development of contralateral pulmonary hypertension. The use of prosthetic tubes should be avoided because reoperation is still needed to change the graft for a bigger size up to adult size. Revascularization by using the autologous tissue or autologous pericardium may obtain a preferred result. The neo-PA stenosis certainly will need to be addressed in the long-term follow-up.

## Data Availability

Applicable.

## Abbreviations

CT: Computed tomography
NI: Nakata index
LPA: Left pulmonary artery
MPA: Main pulmonary artery
PA: Pulmonary artery
RPA: Right pulmonary artery
TOF: Tetralogy of Fallot
UAPA: Unilateral absence of a pulmonary artery
